# Contrast-induced Acute Kidney Injury in Patients with Liver Cirrhosis: A Retrospective Analysis

**DOI:** 10.7759/cureus.2707

**Published:** 2018-05-29

**Authors:** Zain Ul Abideen, Syed Nayer Mahmud, Mohammad Salih, Ali Arif, Furqan Ali, Amna Rasheed, Muhammad Zafran

**Affiliations:** 1 Nephrology and Renal Transplant, Royal Cornwall Hospital, Truro, GBR; 2 Nephrology, Shifa International Hospital, Islamabad, PAK; 3 Gastroenterology and Hepatology, Shifa International Hospital, Islamabad, PAK; 4 Department of Internal Medicine, Shifa International Hospital, Islamabad, PAK; 5 Respiratory Department, Royal Cornwall Hospital, Truro, GBR

**Keywords:** contrast induced nephropathy, acute kidney injury, hepatic cirrhosis, liver cirrhosis

## Abstract

Contrast-induced acute kidney injury (CI-AKI) has been studied less extensively in patients with liver cirrhosis (LC). It is unclear whether the presence of severe liver disease is actually a predisposing factor for CI-AKI. Liver cirrhosis is extremely common in Pakistan and is attributed to the high prevalence of chronic viral hepatitis. Patients with LC often undergo contrast-enhanced computed tomograms (CECT) for various diagnostic and therapeutic purposes, and there have been concerns regarding them being at risk for CI-AKI. The available literature on this topic is scanty, and no study has been conducted in Pakistan. The purpose of this study, therefore, was to determine the frequency of CI-AKI in patients with LC undergoing CECT and to determine any significant predispositions. We retrospectively analyzed the records of 470 LC patients at our center. The frequency of CI-AKI in our study was 5.1%. A higher mean model for end-stage liver disease (MELD), MELD including sodium (MELD-Na), and Child-Pugh (CP) scores was significantly associated with developing CI-AKI (p<0.05). Patients with CI-AKI also had a significantly higher mean international normalized ratio (INR) and serum bilirubin levels, with lower mean venous bicarbonate and serum sodium levels (p<0.05). Our results show that patients with a more advanced liver disease and poorer synthetic function are increasingly susceptible to developing CI-AKI. Further studies can investigate the role of bicarbonate therapy in preventing CI-AKI in LC.

## Introduction

Acute kidney injury (AKI) is a common occurrence in patients with LC. Approximately 20% of hospitalized LC patients have an AKI [1-2]. It is associated with increased morbidity and mortality in these patients and early recognition and treatment are important. The prevalence of AKI in LC varies greatly and various factors are implicated. Intravenous contrast (IC) administration is a known predisposing factor for AKI [1,3]. Patients with LC often undergo a variety of contrast imaging for diagnostic and therapeutic purposes. These are usually for the diagnosis or follow-up of hepatocellular carcinoma or for pre-transplant evaluation [1]. This has raised a concern in recent years regarding contrast-induced acute kidney injury (CI-AKI) in LC patients. Although CI-AKI has been studied relatively well in patients undergoing cardiac interventions, the data regarding patients with LC is scanty. Liver cirrhosis may cause renal hypoperfusion because of hypovolemia, arterial hypotension, and the activation of different neurohormonal systems. This may theoretically place them at risk for developing CI-AKI [1]. The few studies done on this aspect have variable but interesting results. No study has been conducted in Pakistan, which has one of the highest prevalences of viral hepatitis and LC in the world [4]. With the advent of liver transplantation at our center, the number of contrast radiological procedures has also greatly increased in this patient population. With this background, we strived to determine the frequency of CI-AKI in LC patients and to identify any predispositions for its occurrence.

## Materials and methods

We conducted a retrospective study at Shifa International Hospital from January 2017 to August 2017. The approval of the institutional review board and the ethics committee was sought prior to starting the study. We retrospectively examined the case notes, blood investigations, and imaging for LC patients who had a CECT scan between 2013 and 2017. A total of 616 patients were identified and underwent further screening through our inclusion and exclusion criteria.

Data were collected on designed proformas by the study team at Shifa International Hospital. Patients were excluded from the study if they had any of the following: known diagnosis of chronic heart failure, chronic kidney disease (CKD), acute kidney injury (AKI), or sepsis from any source/cause at the time when they had CECT, the presence of gastrointestinal (GI) bleeding, spontaneous bacterial peritonitis, recent (<72 hours) therapeutic paracentesis, and the concomitant use of non-steroidal anti-inflammatory drugs (NSAIDs). Patients who received diuretics and angiotensin-converting enzyme inhibitors (ACEI) or angiotensin receptor blockers (ARBs) on the day of contrast administration were also excluded. Using the above criteria, a total of 616 patients were initially assessed. One hundred and forty-six patients were excluded, and 470 underwent further analysis.

The date of the first CECT was noted and a diagnosis of cirrhosis was confirmed from the scan and patient notes. The amount of intravenous contrast administered was noted; this was a standard at 1.2 ml/kg of iopromide (Ultravist) used at our institution. The following variables were documented before IC administration: serum albumin, international normalized ratio (INR), liver enzymes, serum sodium, and venous bicarbonate. The serum creatinine was noted before and 48 hours post-IC exposure. The estimated glomerular filtration rate (eGFR) was worked before and after IC exposure using the modification of diet in renal disease 4 (MDRD 4) formula. CI-AKI was defined according to the kidney disease improving global outcome (KDIGO) criteria as a rise in the serum creatinine by ≥ 0.3 mg/dL (≥26.5 µmol/L) within 48 hours [5].

The presence of diabetes mellitus, etiology of LC, and presence of hepatocellular carcinoma (HCC) was also noted. Data were entered and analyzed using the Statistical Package for the Social Sciences (SPSS) version 23 (IBM Corp., Armonk, NY, US). Categorical variables were expressed as frequencies and percentages, while continuous variables were expressed as means and standard deviations. Categorical variables were compared using the chi-square test, while for continuous variables, the independent sample t-test was employed. A p-value of less than 0.05 was considered significant.

## Results

A total of 616 patients were initially considered for data collection. One hundred and forty-six patients were excluded on the basis of our exclusion criteria. Four hundred and seventy patients were included in the study for data collection and analysis. The general characteristics of this study cohort are illustrated in Table 1. Twenty-four patients met the criteria for CI-AKI. The frequency of CI-AKI in this study population was 5.1%.

**Table 1:** The general characteristics of patients in the study (n=470) Abbreviations: IC - Intravenous Contrast; MELD - Model for End Stage Liver Disease; CP - Child-Pugh; INR - International Normalized Ratio; ALT - Alanine Transaminase; eGFR - Estimated Glomerular Filtration Rate.

Variable	Value
Mean Age (years)	49.37 ± 9.58
M/F	391(83.2%)/79 (16.8%)
Mean Volume of IC (ml)	106.29± 21.39
Mean MELD-Na score	23.48 ± 13.51
Mean MELD score	17.29 ± 5.26
Mean CP score	9.29 ± 1.81
Mean Serum Albumin (g/dL)	2.64±0.58
Mean Serum Sodium (mmol/L)	129.72 ± 6.40
Mean INR	1.57 ± 0.44
Mean Serum Bilirubin (mg/dL)	6.96 ± 9.27
Mean Venous Bicarbonate (mmol/L)	20.55 ±3.64
Mean ALT (U/L)	55.48 ±36.26
Mean eGFR before IV contrast (ml/min)	94.45 ± 27.67
Diabetes Mellitus	151/470 (32.1%)
Hepatocellular Carcinoma	143/470 (30.1%)

The differences between the CI-AKI and No CI-AKI are detailed in Table 2.

**Table 2:** A comparison of the CI-AKI and No CI-AKI groups Abbreviations: IC - Intravenous Contrast; MELD - Model for End Stage Liver Disease; CP - Child-Pugh; INR - International Normalized Ratio; ALT - Alanine Transaminase; eGFR - Estimated Glomerular Filtration Rate.

Variable	CI-AKI	No CI-AKI	P value
Mean Age (years)	48.67 ± 4.59	49.41 ± 9.78	0.482
M/F	16/8	375/71	0.528
Mean Volume of IC (ml)	103.00 ± 15.44	106.46 ± 21.67	0.305
Mean MELD sodium score	28.67 ± 3.47	23.17 ± 13.82	0.02
Mean MELD score	21.67 ± 6.47	17.04 ± 5.52	0.001
Mean CP score	10.00 ± 1.67	9.26±1.80	0.01
Mean Serum Albumin (g/dL)	2.65 ± 0.50	2.64 ± 0.58	0.921
Mean Serum Sodium (mmol/L)	124.00 ± 1.67	130.05 ± 6.41	0.001
Mean INR	2.00 ± 0.58	1.55 ± 0.41	0.001
Mean Serum Bilirubin (mg/dL)	8.99 ± 7.50	6.85 ± 9.35	0.02
Mean Venous Bicarbonate (mmol/L)	18.00 ± 3.10	20.65 ± 3.63	0.004
Mean ALT (U/L)	47.50 ± 22.20	55.83 ± 36.73	0.173
Mean eGFR before IV contrast (ml/min)	82.29 ± 19.03	95.11 ± 27.93	0.027
Diabetes Mellitus	3/24(12.5%)	148/446 (33.2%)	0.160
Hepatocellular Carcinoma	0 /24 (0%)	143/446 (32%)	0.207

On statistical analysis, patients with CI-AKI had significantly worse mean MELD, MELD-Na, and Child-Pugh scores (p <0.05). Patients with CI-AKI had statistically significant higher mean INR and serum bilirubin levels (p<0.05). They also had statistically significant lower mean serum sodium and venous bicarbonate levels (p<0.05). The mean eGFR was significantly lower in the CI-AKI group (p<0.05).

## Discussion

Acute kidney injury can develop via numerous mechanisms in patients with liver cirrhosis. The hemodynamic abnormalities and bacterial translocation from the intestines are predisposing factors for this. In these conditions, IC may simply serve as a trigger for AKI [2]. In order to obtain more authentic results, we strived to exclude conditions that are associated with AKI in liver cirrhosis. This is detailed in the methodology section.

The frequency of CI-AKI in our study was 5.1%. This is relatively lower as compared to some previous studies done on this particular topic; a previous study by Safi et al. concluded a frequency of 17.9%. Another recent study found that 8.8% of the LC patients developed CI-AKI. An older study concluded a frequency of 25% [1-3]. All of these were retrospective studies and the differing incidences may be because of different exclusion criteria and patient populations. The studies with the higher incidence had a more relaxed exclusion criterion, which may have affected the overall results. Our lower frequency may be the result of more stringent exclusion criteria.

We did not find any association between CI-AKI and age or gender. The former is supported by previous studies [1-3]. A few past studies have concluded a relationship between CI-AKI and a postmenopausal state in women. Although we did not find any association to gender, this may be because most of our patients were young and probably not in the menopausal category. It is postulated that after menopause the beneficial effect of estrogen against cardiovascular and renal disease is lost, making individuals more prone to kidney insults [1,6].

The CP and MELD scores are widely used for assessment of prognosis in LC [7]. The MELD-Na score additionally incorporates serum sodium levels into the MELD score [8]. The higher these scores, the worse the prognosis and mortality in advanced liver disease. Table 2 summarizes an important finding of our study; patients who developed CI-AKI had worse CP, MELD, and MELD-Na scores as compared to those who did not have CI-AKI. This generally means that the former had more severe liver dysfunction and a worse prognosis. This finding is further supported by the fact that the mean INR and serum bilirubin levels were higher in the CI-AKI group. A higher INR indicates a poorer synthetic function of the liver. This has not been a consistent finding in previous studies. The association with a worse INR has been described before [2]. These results may have important implications. Patients with severe cirrhosis and worse prognostic scores may be more susceptible to CI-AKI; this again may be due to a combination of an abnormal circulatory and hormonal milieu as described earlier.

Patients with CI-AKI in our study had a statistically significant lower mean serum sodium level as compared to those who did not have CI-AKI. This is depicted in Table 2. This finding seems to be more consistent in past studies. Hyponatremia is an independent mortality predictor of advanced liver disease. Studies have found it to be associated with increased mortality both before and after liver transplantation [9]. Hyponatremia in liver cirrhosis is usually dilutional or hypervolemic. This is because of the activation of the renin-angiotensin and aldosterone system and sympathetic nervous system, and the non-osmotic release of anti diuretic hormone (ADH). This results in increased tubular absorption of water and consequent hyponatremia [9]. Therefore, more severe hyponatremia may be a marker of severe liver dysfunction [10]. The fact that our patients with CI-AKI had lower serum sodium further complements the earlier findings of CI-AKI occurring in patients with severe liver disease.

None of the retrospective studies done on this topic have investigated the association of venous bicarbonate levels with CI-AKI. The KDIGO guidelines mention the use of intravenous sodium bicarbonate in patients at high risk for CI-AKI [5]. There is some evidence that low serum bicarbonate levels may predispose to AKI [11]. We found statistically lower venous bicarbonate levels in the CI-AKI group. Since we excluded patients with sepsis and other conditions that may lower the serum bicarbonate, these results may have important implications. Further studies could investigate the role of intravenous sodium bicarbonate in the prevention of CI-AKI in patients with LC. A past study found no difference between intravenous albumin and sodium bicarbonate in preventing CI-AKI in patients with liver cirrhosis and chronic kidney disease. It did, however, conclude a lower incidence of CI-AKI in patients receiving these agents [12]. This study was, however, limited by its sample size and methodology.

Interestingly, patients with CI-AKI in our study had a statistically significant lower mean eGFR as calculated by the MDRD 4 equation. This was still however in the normal range and none of our patients had CKD. All patients with CKD were excluded from the study as it is a known predisposing factor for CI-AKI. Since patients with CI-AKI had relatively severe liver dysfunction as explained earlier, the lower mean eGFR may be a consequence of this. This is also supported by the lower mean serum sodium levels in the CI-AKI group. As explained earlier, the interplay of circulatory and hormonal factors may have been more severe in this group resulting in a relatively lower kidney function.

## Conclusions

The frequency of CI-AKI in this large Asian cohort of liver cirrhotic patients was 5.1%. Worse liver prognostic scores, hyponatremia, and lower venous bicarbonate levels were associated with the development of CI-AKI. Future studies may target the role of bicarbonate therapy in patients with severe liver dysfunction undergoing intravenous contrast administration.
